# Case Report: Nonsurgical management of painless cervical motor radiculopathy

**DOI:** 10.3389/fresc.2025.1695867

**Published:** 2025-11-20

**Authors:** Yong Ho Lee

**Affiliations:** Leadmpain Clinic, Daegu, Republic of Korea

**Keywords:** cervical radicular pain, motor paralysis, epidural steroid block, nonsurgical treatment, transforaminal block, motor recovery

## Abstract

**Background:**

Painless motor radiculopathy is a rare clinical entity, accounting for approximately 5%–8% of cervical radiculopathy cases. While current guidelines recommend early surgical intervention for patients with severe motor deficits, the efficacy of nonsurgical management remains unclear.

**Case presentation:**

We present a case of a 67-year-old man with sudden onset of right shoulder motor paralysis [Medical Research Council (MRC) grade 3-] without pain. MRI revealed multilevel foraminal stenosis at C5–C7–T1 and central disc protrusion at C4–5, without cord compression. The patient elected nonsurgical management and received four epidural steroid injections (two interlaminar and two transforaminal) over five weeks. Motor function progressively improved, reaching MRC grade 5-, and shoulder range of motion was fully restored. No adverse effects occurred.

**Conclusion:**

This case demonstrates that epidural steroid injections can be an effective alternative to surgery for selected patients with painless cervical motor radiculopathy and severe motor dysfunction. Individualized treatment strategies should be considered, and further research is warranted to establish optimal management approaches.

## Introduction

Cervical radiculopathy is a common neurological disorder characterized by varying degrees of pain, sensory disturbances, and motor weakness resulting from nerve root compromise, most often due to degenerative changes, herniated discs, or foraminal stenosis (1, 2). Although pain typically predominates, a minority of patients present with severe motor weakness in the absence of pain, known as painless motor radiculopathy (1, 2). This rare clinical entity accounts for only 5%–8% of all cervical radiculopathy cases, posing unique diagnostic and therapeutic challenges.

Patients with profound initial weakness (MRC grade 3 or below) are at risk of delayed diagnosis and permanent neurological impairment. Current guidelines, including those from the American Association of Neurological Surgeons and the North American Spine Society, recommend early surgical intervention for severe motor deficits, multilevel foraminal stenosis, or progressive neurological deterioration (3, 4). These recommendations are based on concerns about irreversible loss of function and the assumption that conservative management is less effective in severe cases.

However, the evidence supporting these guidelines is limited, especially for patients with painless cervical radiculopathy. Recent reports have described meaningful recovery with conservative treatments, such as epidural steroid injections, even in cases of severe motor dysfunction (5–8). Nevertheless, complete functional recovery without surgical intervention in patients with severe motor deficits and no pain remains exceedingly rare.

To our knowledge, few reports exist of patients with acute, painless, severe motor paralysis due to cervical radiculopathy achieving rapid and complete recovery through conservative management alone. This case is unique in that the patient presented with profound motor weakness (MRC grade 3) of the right shoulder, yet experienced full restoration of function within one month, without surgery. Such an outcome is rare and challenges the prevailing paradigm that early surgery is mandatory for severe motor deficits.

Here, we report a very rare case of acute, painless, severe motor paralysis of the right shoulder due to cervical radiculopathy that achieved complete recovery with conservative management, including epidural steroid injection. This case highlights the potential for individualized, nonsurgical treatment to yield excellent outcomes even in patients typically considered surgical candidates. Our experience provides important clinical implications for future practice and research, suggesting that conservative management may be an effective and safe option for selected patients with severe, painless motor radiculopathy.

## Case presentation

A 67-year-old male farmer presented to our clinic with a sudden onset of profound motor deficit in his right shoulder, which developed overnight without any preceding trauma or warning signs. His medical history was notable only for hypertension, managed with antihypertensive medication. He denied any history of chronic illnesses, additional medication use, or prior surgeries. There were no recent episodes of trauma, infection, or systemic symptoms.

Remarkably, the patient reported complete absence of pain, sensory disturbance, or paresthesia in the affected limb. He had no factors aggravating or relieving pain, as his chief complaint was isolated, painless motor weakness. Functionally, he was unable to lift his right arm above shoulder level, resulting in significant limitations in daily life. He could not wash his face, comb his hair, or perform overhead tasks, severely impacting his ability to carry out essential farming activities. The timing of symptom onset coincided with peak farming season, making any interruption to his work especially detrimental to his livelihood.

On neurological examination, there was marked motor weakness in the right shoulder, graded as MRC 3- in flexion and abduction, and MRC 4 in extension. The range of motion (ROM) of the right shoulder was limited to 60 degrees. Sensory examination was unremarkable, and motor and sensory functions of the right elbow, wrist, and hand were normal. Muscle strength was assessed using the modified British Medical Research Council (MRC) scale (9).

**Figure 1:**
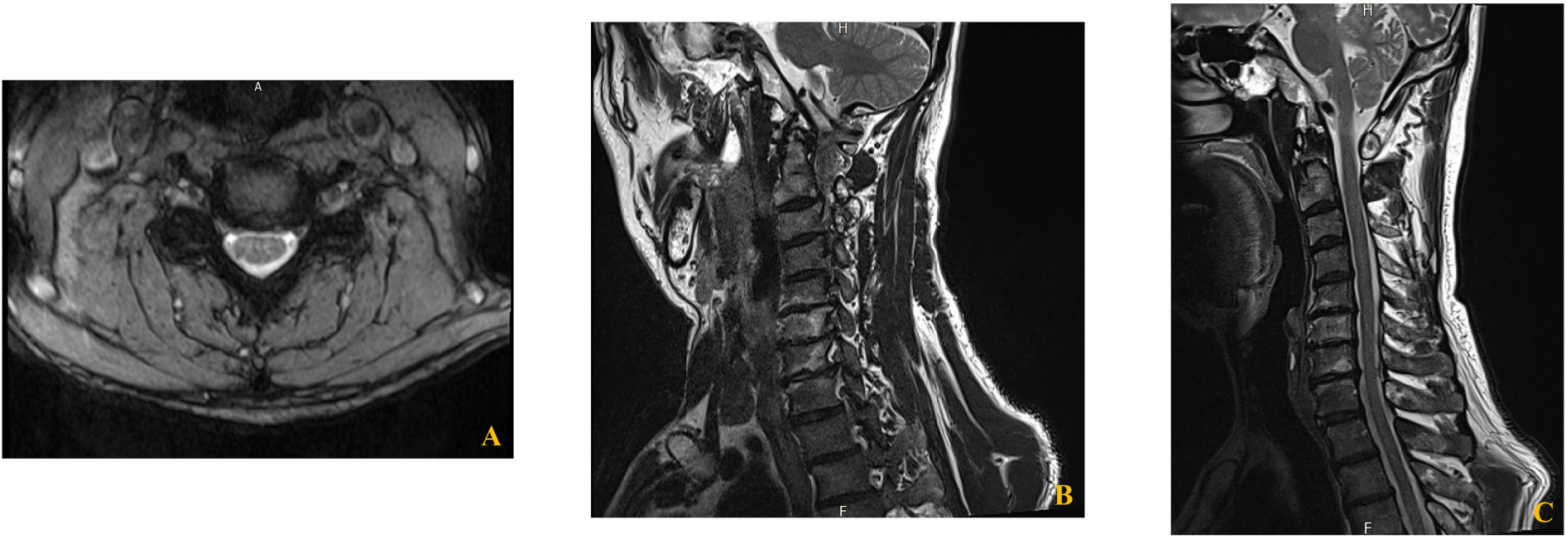
Cervical spine MRI demonstrating right sided foraminal stenosis from C5 to T1. **(A)** Axial view showing cervical spinal canal and neural foraminal anatomy. **(B)** Right oblique view with visible cervical vertebrae (C4–C7) demonstrating right-sided foraminal stenosis. **(C)** Sagittal view showing the cervical spine alignment and neural foraminal narrowing.

Radiographic evaluation included cervical spine x-rays, which showed multilevel degenerative changes such as reduced intervertebral disc height and osteophyte formation (10, 11). Cervical spine MRI revealed bilateral foraminal stenosis, most pronounced at C5–6–7-T1 on the right side, and a central disc protrusion at C4–5 (Figure 1) (12). There was no evidence of focal nerve root compression or abnormal signal change in the spinal cord.

Given the absence of pain, sensory symptoms, and any signs suggestive of infection or systemic inflammation, laboratory workup to exclude inflammatory etiologies was not performed. Our clinic is a primary care facility with limited laboratory resources, and the patient's clinical presentation did not warrant additional testing. Furthermore, his condition gradually improved with conservative management, and additional laboratory tests would have incurred unnecessary costs and delays. Referral for further laboratory evaluation was available if indicated.

After a thorough discussion of risks, benefits, and treatment options, the patient strongly preferred to avoid surgical intervention due to his occupation and social circumstances. As a farmer during the busiest season, he could not afford the downtime and potential complications associated with surgery, as it would directly threaten his livelihood. Therefore, a nonsurgical treatment plan was selected, prioritizing conservative management tailored to his clinical and social needs.

## Intervention

**Figure 2:**
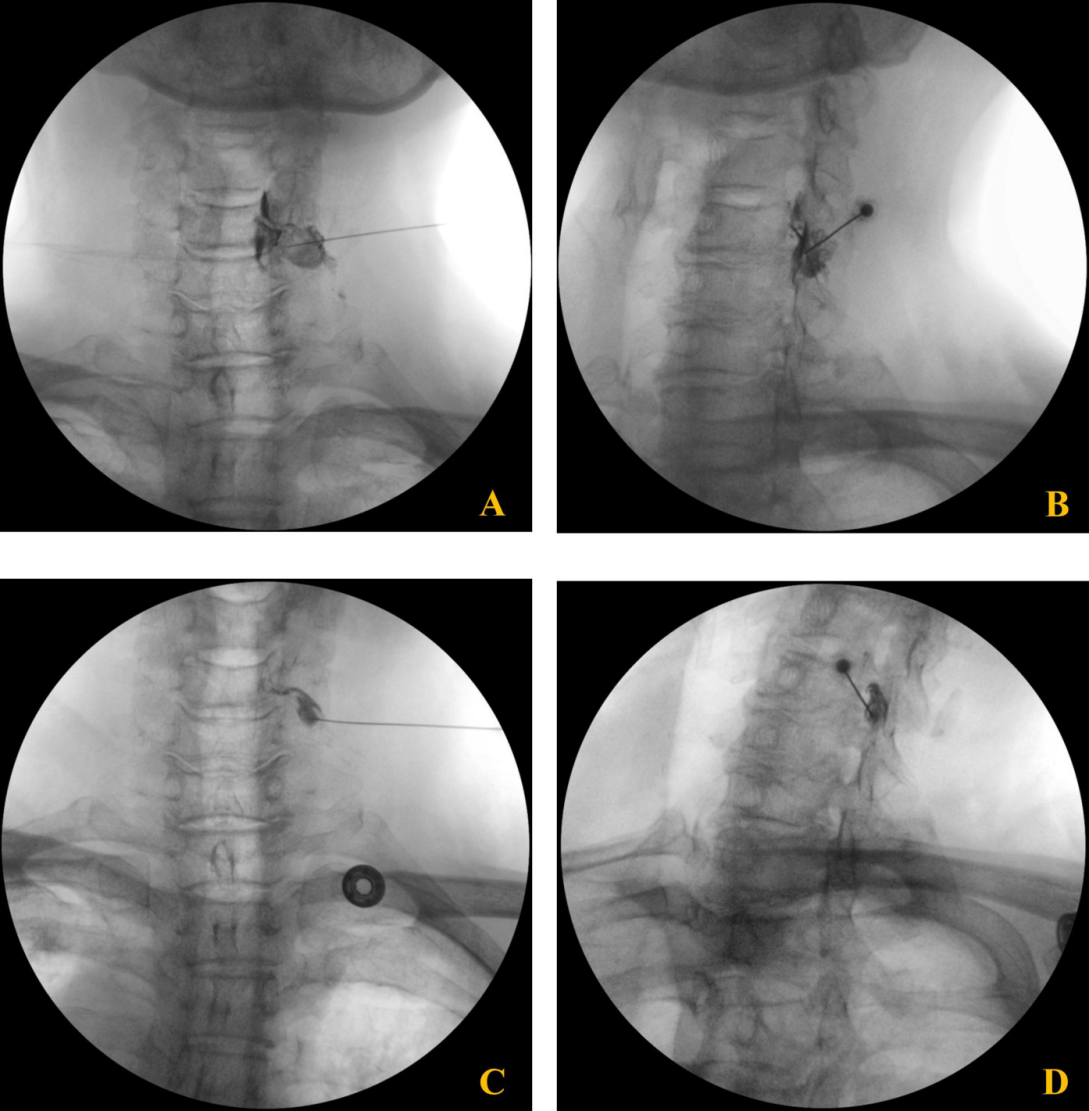
Fluoroscopic images of C6 cervical transforaminal epidural steroid injection. **(A,B)** Anteroposterior **(A)** and oblique **(B)** views of the second injection showing needle placement and contrast spread at the C6 neural foramen. **(C,D)** Anteroposterior **(C)** and oblique **(D)** views of the third injection demonstrating proper needle positioning and contrast distribution along the C6 nerve root pathway.

Over a five-week period, the patient received four epidural steroid injections under C-arm fluoroscopic guidance. The interlaminar approach was performed at the C6–7 level using a paramedian technique, directing the needle toward the right side for optimal drug delivery. The transforaminal approach targeted the right C6 nerve root by advancing the needle toward the C5/6 intervertebral foramen (Figure 2). Each injection consisted of dexamethasone palmitate (4 mg) (13), hyaluronidase (1,500 IU) (14), and 0.1% lidocaine (5 mL) (15). The patient was evaluated at regular intervals (4 days, 2 weeks, 4 weeks, and 5 weeks), with assessments of motor strength, shoulder ROM, and subjective symptom improvement. The clinical course, including changes in muscle strength and range of motion, is summarized in Table 1.

**Table 1:** Clinical progress of a patient.

Time	Motor function (MRC grade)	ROM 0–180)	Patient's subjective recovery	Procedure
First visit	3-	60	0%	CESI (IL)
Day 4	3-	60	0%	CESI (TF, C6)
Week 1	3-	90		
Week 2	3-	90	50%	CESI (TF, C6)
Week 3	3-	120		
Week 4	4	180	80–90%	CESI (IL)
Week 5	5-	180		

CESI, cervical epidural steroid injection; IL, interlaminar approach; TF, transforaminal approach; ROM, range of motion; MRC, medical research council.

At each outpatient follow-up, the patient's motor function and shoulder mobility were documented and compiled into a Supplementary Video, demonstrating the sequential recovery process over the five-week treatment period.

After the first interlaminar injection, no significant improvement in motor function was observed at the 4-day follow-up. Following the first transforaminal injection, the patient reported approximately 50% improvement in motor strength and restoration of shoulder ROM to 90 degrees at the 2-week visit. A second transforaminal injection was administered at this time. By the 4-week follow-up, shoulder ROM had recovered to 180 degrees, and motor function improved to nearly normal, with only mild residual weakness. A final interlaminar injection was performed, and at the 5-week assessment, the patient's motor strength reached MRC grade 5-, with full functional recovery and no limitations in daily activities. Mild fatigue in the right arm was noted during strenuous activity, but there were no adverse effects related to the procedures.

The patient was prescribed pregabalin (75 mg twice daily) (16) and initiated on exercise therapy (17). He was scheduled for continued follow-up to monitor for recurrence and long-term outcomes.

## Ethical consideration

This study was approved by the e-IRB Ethics Committee (IRB P01-202507-01-022), and written informed consent for publication was obtained from the patient. All personal identification information has been de-identified.

At the final follow-up, the patient reported high satisfaction with nonsurgical treatment, complete recovery of shoulder function, and a successful return to daily activities and work without limitation. He expressed gratitude for the individualized care and the opportunity to avoid surgery.

## Discussion

This case presents a rare and clinically significant scenario of severe, painless motor radiculopathy secondary to multilevel cervical foraminal stenosis, which was successfully managed with conservative treatment alone. Unlike the typical manifestation of cervical radiculopathy—where pain predominates and guides both diagnosis and management, our patient exhibited isolated, profound motor weakness without any pain or sensory disturbance. Such cases are exceedingly uncommon and are rarely discussed in literature, which predominantly focuses on painful radiculopathy.

The novelty and clinical importance of this case are multifaceted.

First, it directly challenges the prevailing paradigm that early surgical intervention is mandatory for patients with severe motor deficits, particularly in the setting of multilevel cervical stenosis. Major neurosurgical and spine society guidelines currently advocate for prompt surgery to prevent irreversible neurological sequelae in such patients (3, 4). However, these recommendations are largely based on studies involving patients with significant pain or progressive neurological decline, and there is a notable lack of evidence addressing the optimal management of painless, yet severe, motor radiculopathy. Our case demonstrates that, in carefully selected individuals, nonoperative management can result in rapid and complete functional recovery, even in the presence of profound motor impairment.

Second, this case highlights a critical gap in clinical practice: the absence of clear, evidence-based guidelines for treating patients who present with painless but disabling motor deficits due to cervical radiculopathy. Such atypical presentations can lead to diagnostic uncertainty, delayed intervention, or unnecessary surgery. Our experience underscores the value of individualized, patient-centered decision-making—taking into account not only clinical and radiological findings but also the patient's occupational and social circumstances. In this instance, our patient's role as a farmer during the peak agricultural season precluded surgical intervention, making conservative management not only a medical but also a socioeconomic necessity.

From a therapeutic perspective, this case illustrates the potential efficacy of targeted epidural steroid injections, in combination with physical therapy and pharmacologic support, for restoring motor function in patients with multilevel cervical foraminal stenosis (5–8). The use of both interlaminar and transforaminal injection techniques enabled comprehensive delivery of anti-inflammatory agents to the affected nerve roots, likely facilitating rapid and sustained neurological recovery.

Notably, the adjunctive use of hyaluronidase in epidural injections may have contributed to the favorable outcome in this case (14). Hyaluronidase, an enzyme that hydrolyzes hyaluronic acid in the extracellular matrix, enhances the permeability of tissues and promotes more uniform distribution of injected medications. In pain medicine, although not routinely used, hyaluronidase has been reported to improve drug delivery to the target site, reduce local fibrosis, and potentially decrease adhesions around nerve roots. By facilitating the dispersion of corticosteroids and reducing perineural scarring, hyaluronidase may augment the anti-inflammatory and analgesic effects of epidural injections, especially in cases with chronic inflammation or previous interventions. Further studies are warranted to clarify its role and establish standardized protocols for its use in spinal interventions.

In addition to epidural steroid injections, various other nonsurgical modalities have been explored in the management of cervical radiculopathy. These include regenerative therapies such as mesenchymal stem cell injections or platelet-rich plasma (PRP), ozone therapy, radiofrequency ablation, and cryotherapy. While these approaches have shown promise in selected cases and are increasingly utilized in pain medicine, their efficacy and safety profiles require further validation through robust clinical trials. Regardless of the chosen intervention, physical therapy and rehabilitation remain essential components of comprehensive care, supporting both short- and long-term functional recovery (17).

Importantly, this case expands the current understanding of cervical radiculopathy by demonstrating that the absence of pain does not exclude the possibility of significant neurological compromise—or the potential for recovery with conservative therapy (2, 6). It emphasizes the need for careful clinical assessment and shared decision-making, rather than rigid adherence to guideline-based recommendations. For clinicians, this case offers a practical approach: thorough radiological evaluation to exclude cord compression, judicious use of epidural steroid injections, and close monitoring of motor recovery (10–12). By documenting sequential improvements in muscle strength and function, we provide objective evidence that conservative management can be both safe and effective in selected patients, potentially avoiding the risks and costs associated with surgery.

Nevertheless, this report has several limitations. As a single-case observation, its findings cannot be generalized to all patients with painless motor radiculopathy. The lack of long-term follow-up also precludes assessment of sustained outcomes or late relapse. Further research—including prospective studies comparing surgical and conservative approaches, as well as investigations into optimal injection techniques and rehabilitation protocols—is warranted to better define the role of nonsurgical management in this unique patient population.

In conclusion, this case broadens the clinical spectrum of cervical radiculopathy and provides valuable insights into the management of painless motor paralysis. It underscores the importance of individualized care and suggests that, with careful patient selection and close follow-up, conservative therapy may be a viable and effective alternative even in severe cases.

## Data Availability

The original contributions presented in the study are included in the article/Supplementary Material, further inquiries can be directed to the corresponding author.
